# Influence of the Type of Basal Insulin and Other Variables on Clinical Outcomes in Children with Newly Diagnosed Type 1 Diabetes

**DOI:** 10.1155/2014/758343

**Published:** 2014-02-06

**Authors:** Ruth M. Garrison, Jeremy L. Johnson, Michelle E. Condren, Kevin C. Farmer, David H. Jelley

**Affiliations:** ^1^Ambulatory Care, Veterans Affairs Medical Center, 921 NE 13th Street, Oklahoma City, OK 73104, USA; ^2^Department of Pharmacy, Clinical and Administrative Sciences, University of Oklahoma College of Pharmacy, 4502 East 41st Street, Suite 1H05, Tulsa, OK 74135, USA; ^3^Department of Pediatrics, University of Oklahoma School of Community Medicine, 4502 East 41st Street, Tulsa, OK 74135, USA; ^4^Department of Pharmacy, Clinical and Administrative Sciences-Tulsa, University of Oklahoma College of Pharmacy, P.O. Box 26901, Oklahoma City, OK 73126, USA; ^5^Harold Hamm Oklahoma Diabetes Center, Tulsa Program, University of Oklahoma School of Community Medicine, 4502 East 41st Street, Tulsa, OK 74135, USA

## Abstract

*Background*. Basal insulin detemir and glargine each have characteristics that may make them a superior choice in children and adolescents with type 1 diabetes, but there is a paucity of data on glycemic results in this population. *Objective*. Examination of variables associated with achievement of HbA1c goal in children and adolescents with newly diagnosed type 1 diabetes. *Methods*. The primary outcome, factors associated with achievement of HbA1c goal, was examined in a retrospective chart review. Variables, including type of basal insulin, were collected during the first year of diagnosis of patients in a pediatric diabetes clinic. Secondary outcomes included change in HbA1c, severe hypoglycemic events, and episodes of DKA. *Results*. 94 patients were included in the study. HbA1c at diagnosis was found to be a significant predictor of achievement of goal at 3 months (*P* = 0.002) and of change in HbA1c at 3 and 12 months (*P* < 0.001 for each). Severe hypoglycemia and episodes of DKA were uncommon. *Conclusions.* Choice of basal insulin was not found to be a predictor of achieving HbA1c goal or of change in HbA1c over the course of the first year of diagnosis with type 1 diabetes.

## 1. Introduction

In patients newly diagnosed with type 1 diabetes, clinicians have three choices of basal insulin: NPH or one of two insulin analogs, glargine and detemir. While NPH is the traditional choice, the insulin analogs better mimic natural insulin secretion. In a meta-analysis, Monami and colleagues found that the use of glargine and detemir resulted in decreased hemoglobin A1c (HbA1c) and a reduced risk of nocturnal and severe hypoglycemia when compared to insulin NPH [1]. The American Diabetes Association recommends the use of insulin analogs in many patients with type 1 diabetes, particularly those in whom hypoglycemia has been a complicating factor [2].

The choice of long-acting analog in newly diagnosed patients with type 1 diabetes is less clear. Several studies have measured pharmacokinetic and dynamic endpoints. A study in patients with type 1 diabetes demonstrated that while the activity of glargine and detemir was initially similar, after 12 hours detemir's effects decreased significantly, and the average end of action was 17.5 hours compared to glargine's 24 hours [3]. Detemir has been found to have a dose-dependent duration, with lower doses having a shorter duration of action. It is also possible that the duration of action will be attenuated in children and adolescents with type 1 diabetes [4]. However, Heise and colleagues found that, when compared to insulin NPH and glargine, detemir had decreased pharmacodynamic within-subject variability over 24 hours, measured by the area under the glucose infusion rate curve (GIR-AUC) using a euglycemic glucose clamp procedure [5]. Another study examined the AUC of insulin concentration in children and adolescents with type 1 diabetes. It found significantly less within-subject variability with detemir in comparison to glargine; however the measurements only continued for 16 hours after the dose and perhaps did not reflect the variable duration of detemir [6].

The literature conflicts when comparing the clinical outcomes of glargine and detemir. Pieber and colleagues compared once-daily glargine to twice-daily detemir in adults and found a similar change in HbA1c and no difference in overall within-subject variation or hypoglycemic events. They found that within-subject variation in predinner plasma glucose was significantly higher with glargine, thought to be due to the dose beginning to wear off. Although fasting plasma glucose was significantly lower with glargine, the risk of severe and nocturnal hypoglycemias was increased [7]. In contrast to this study lie the findings of Yenigun and colleagues, who performed an observational study examining adults with types 1 and 2 diabetes switching from glargine once or twice daily to detemir once daily. This study found that detemir significantly decreased HbA1c, fasting plasma glucose, and variability in fasting plasma glucose compared to glargine. In addition, it found a reduction in hypoglycemia, including severe and nocturnal events, with detemir [8]. Yet, when Kabadi retrospectively examined adults with type 1 diabetes undergoing conversion from glargine once daily to detemir twice daily, an increase in HbA1c was found despite a higher daily dose of detemir [9]. Unfortunately, none of these studies of clinical outcomes were performed in newly diagnosed patients with type 1 diabetes. Therefore, this study proposes to examine this group of patients and compares the use of basal insulin in patients achieving HbA1c goal in contrast to those who do not.

## 2. Methods

The study was performed as a retrospective chart review at a pediatric diabetes clinic. Inclusion criteria were diagnosis of type 1 diabetes, age < 21 years, active participation in clinic during the first year of diagnosis, and use of detemir or glargine as basal insulin. Exclusion criteria were concomitant diagnosis or drug use that compromised control of diabetes, pregnancy, enrollment in interventional clinical trials, or concomitant use of antidiabetic drugs other than insulin.

Patients who were diagnosed from June 2007 to December 2009 were reviewed for eligibility for this study. Patients were categorized into two groups for comparison on the basis of having achieved goal HbA1c at one year from diagnosis. Goal HbA1c was defined as <7.5% as recommended by the International Society for Pediatric and Adolescent Diabetes [10]. HbA1c was determined by use of the Afinion AS100 Analyzer System. The primary outcome was the examination of factors associated with achieving or not achieving goal HbA1c at 12 months. Secondary outcomes were change in HbA1c and number of episodes of severe hypoglycemia and diabetic ketoacidosis. Severe hypoglycemia was defined as a hypoglycemic episode requiring the assistance of another to resolve per patient report. Factors that were analyzed included type of basal insulin prescribed, gender, age, type of insurance coverage, type of rapid-acting insulin prescribed, ethnicity, BMI percentile, type of dosing used (set v. flexible), dose by unit/kg, presence of hypothyroidism, completion of education visits, frequency of monitoring, HbA1c at diagnosis, presence of diabetic ketoacidosis at diagnosis per physician report, scores on pre- and posteducation tests, and the person administering injections. The preceding factors are items that may influence the achievement of HbA1c goal. Data was collected at 0, 3, 6, and 12 months after diagnosis, ±1 month to accommodate the flexible timing of follow-up visits. Choice of basal insulin was based on provider's and health benefit provider's preference. Standard dosing practice in the studied clinic was to initiate insulin detemir or glargine at a dose of 0.5–0.75 units/kg/day with aspart or lispro at mealtimes dosed using insulin to carbohydrate ratios and correction factors.

A multivariate logistic regression was used to analyze the factors associated with patients achieving or not achieving the HbA1c goal. A multivariate linear regression was used to analyze the change in HbA1c. Chi-squared analysis was used to compare baseline categorical data, while the independent samples *t*-test was used to compare baseline continuous data. All statistics were performed using SPSS Statistics 17.0.

The protocol was approved by the governing Institutional Review Board and was performed in accordance with the Declaration of Helsinki.

## 3. Results

**Figure 1:**
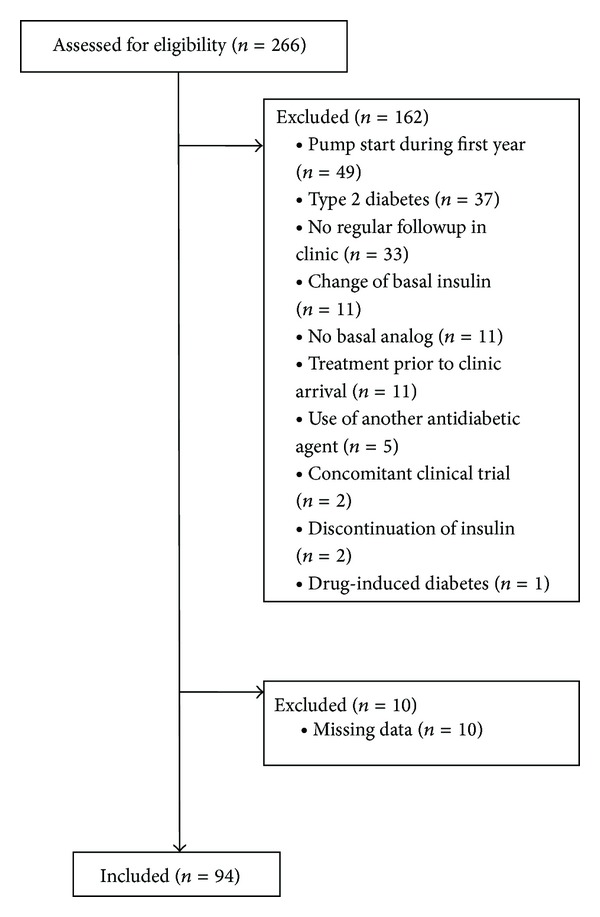
Excluded patients.

A total of 266 patients were seen in the diabetes clinic in the specified time frame. Of these, 104 were qualified for inclusion in this study.  Figure 1 describes the rationale for exclusion. A further 10 patients missed a single followup, resulting in exclusion due to missing data. Therefore, the final number of patients included was 94, 38 in the glargine group and 56 in the detemir group. Two patients had full followup but were missing HbA1c (both in the glargine group, one at three months and one at six months). These patients were excluded from the regression analyses. Baseline characteristics are described in Table 1. Only one significant difference was found between the baseline characteristics of the glargine and detemir groups, that of the use of insulin aspart as rapid acting insulin at baseline (68% of glargine group, 100% of detemir group, *P* < 0.001). Standard practice in the clinic studied is to administer basal insulin once daily; however, two patients on glargine and one patient on detemir received twice daily injections. No patterns emerged in those who were excluded for changing between basal insulin during the first year. The person administering injections was not often recorded at baseline; thus there was too little data to perform statistics on this measure. All other recorded characteristics were not significantly different between groups.

The primary endpoint was achievement of HbA1c goal at 12 months. No difference was found between patients using glargine and those using detemir in the 12-month measure, as well as at 3 months. In the glargine group, 42% (16 of 38) of patients achieved HbA1c goal, while in the detemir group, 55% (31 of 56) of patients achieved HbA1c goal at 12 months, with a *P* = 0.207 (Table 2). At three months, 76% (28 of 37) of patients achieved goal in the glargine group, compared to 68% (38 of 56) in the detemir group (*P* = 0.416). However, at six months, 76% of glargine patients achieved goal (28 of 37) compared to 93% of detemir patients (52 of 56, *P* = 0.019). When logistic regression was performed, the only variable found to be a significant predictor of achievement of goal was HbA1c at baseline, and this variable was only significant at three months (*P* = 0.002). The mean baseline HbA1c for patients achieving goal at 3 months was 11.1% versus 12.9% for those not achieving goal (*P* < 0.001). No significant variables were found at six or 12 months. Choice of basal insulin did not influence achievement of HbA1c goal. A stepwise logistic regression was performed, but again no variable was found to be a significant predictor of achievement of goal.

**Figure 2:**
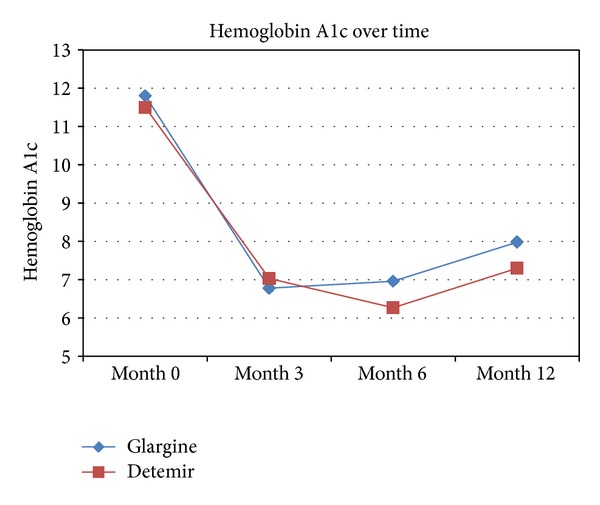
Hemoglobin A1c over time in patients on insulin glargine and detemir.

For the secondary endpoint, change in HbA1c, no significant difference was seen between glargine and detemir during the course of the year. At 3 months, there was a mean decrease of 4.97% in the glargine group, compared to 4.51% in the detemir group (*P* = 0.278). At 6 months, the HbA1c had decreased 4.86% from baseline in the glargine group, and 5.27% from baseline in the detemir group (*P* = 0.444). Finally, at 12 months, the mean decrease from baseline in the glargine group was 3.81% versus 4.24% in the detemir group (*P* = 0.428) (Figure 2). When linear regression was performed, HbA1c at diagnosis was the only variable found to significantly predict the change in HbA1c. A significant difference was seen at three and 12 months for this variable (*P* < 0.001). None of the other variables, including choice of basal insulin, were found to be significant predictors. The *R* ^2^ for 3 months was 0.808 and for 12 months was 0.627, indicating that 80.8% and 62.7% of the variance in the change in HbA1c are explained by the regression model at each respective time point.

Safety measures that were examined as secondary endpoints were episodes of severe hypoglycemia and diabetic ketoacidosis. Very few episodes of either measure were noted. One episode of hypoglycemia was recorded in each group at 3 months, one episode occurred in the glargine group at 6 months versus two episodes in the detemir group, and no occurrences were recorded at 12 months. One episode of DKA appeared in the detemir group and none in the glargine group at 3 months, no episodes occurred at 6 months, and one episode took place in the glargine group, with none in the detemir group, at 12 months. Too few episodes occurred to perform statistical analysis.

## 4. Discussion

The null hypothesis could not be rejected; no difference was found in the use of basal insulin in those who achieved goal HbA1c at 12 months and those who did not. The only significant variable found was HbA1c at diagnosis, with patients achieving goal HbA1c at three months having a lower average HbA1c at baseline. At 6 and 12 months, the effect was no longer evident on achievement of goal. While baseline HbA1c was found to be a significant predictor of change in HbA1c over time, one must use caution in interpreting this data, as the confounding variable and the outcome are both measured using the HbA1c. A patient who presents with a higher HbA1c has a greater potential for improvement in HbA1c upon receiving treatment. Although a significant difference was seen in achievement of HbA1c goal between glargine and detemir at 6 months, this comparison does not take the other confounders into account, and the logistic regression performed at this time point did not reveal basal insulin to be a predictor of achievement of HbA1c goal. One baseline difference was noted between the groups, as the detemir group was significantly more likely to be using insulin aspart as rapid-acting insulin than the glargine group. The reason for this discrepancy is that insulin pens are routinely used in the clinic, and the pen needles that are compatible with detemir pens are also compatible with aspart pens, but not with lispro. Thus, patients who receive detemir almost always receive aspart, whereas patients receiving glargine may receive either aspart or lispro.

The *R* ^2^ represents how much of the variance in the change in HbA1c is predicted by the regression model. Our high *R* ^2^ values indicate that the variables chosen to be included in the model accounted for the majority of the variances seen. There are variables that likely had a contribution to the primary outcome that could not be measured. Foremost of these variables is the honeymoon period. As there is no objective measure of the honeymoon period that could be collected for the purposes of this study, it was acknowledged by the authors that this would influence the primary outcome and was the chief reason for hypothesizing that no difference would be found in the effect of basal insulin on achievement of goal. There are several different definitions of the honeymoon period, but they each encompass control of HbA1c with small amounts of insulin [11], which clearly decreases the influence of the choice of basal insulin on control of diabetes. Although the maximum effect of the honeymoon is typically seen around 1–4 months after diagnosis, and this study examined outcomes until 12 months, it is still impossible to assert how much effect the honeymoon period had on achievement of goal.

Psychological struggles are another potential confounder that could not be easily measured. Issues such as depression and lack of coping skills can adversely affect adherence and therefore diabetes control [12]. Collection of data on psychiatric diagnoses could have resulted in further strengthening of the regression model. One study found that math skills and literacy had an influence on glycemic control [13], but again, in the present study this information was not recorded and could not be obtained from a retrospective chart review. While this study did not find a relationship between frequency of monitoring and achievement of goal, Levine and colleagues found that increased monitoring predicted greater glycemic control [14]. Levine's study observed patients with an average duration of diabetes of five years, which might account for the difference in results, since the honeymoon period would no longer be a confounding factor at that time. Due to its shorter duration of action, detemir is often dosed twice daily. However, in this study once daily dosing was predominately used for both glargine and detemir with similar results. In accordance, Nimri and colleagues found no difference in diabetes control in pediatric patients using once- versus twice-daily detemir dosing [15].

This study was unique in that it examined the question of the influence of the choice of basal insulin, along with other factors, on glycemic control in the first year of diagnosis with type 1 diabetes. While studies that compare detemir and glargine exist, none were conducted in this particular patient population. Levine examined predictors of glycemic control in pediatric type 1 diabetes, but the study was performed prior to the use of basal insulin analogs and therefore did not address our primary question [14]. Kurtoglu and colleagues examined children with type 1 diabetes who switched from insulin NPH or glargine to detemir and found improved HbA1c along with fewer hypoglycemic events [16]. However, the majority of patients had previously been on NPH and the results did not differentiate between those who had been on NPH and glargine prior to conversion to detemir. Both basal analogs have clear advantages over NPH [1]; thus this study does not demonstrate which basal insulin is superior. Insulin detemir and insulin glargine each had perceived advantages in the treatment of children with type 1 diabetes. Glargine has a duration of action independent of dose and should theoretically last longer for young patients taking small doses (<0.4 units/kg/day) than detemir, which has a dose-dependent duration of action [3]. However, two studies found lower within-subject variability with detemir, one of these studies being performed in children [5, 6]. Conflicting data exists as to the HbA1c lowering effect of detemir compared to glargine, with studies finding no difference, a decrease, and an increase [7–9]. Detemir was found, however, to decrease hypoglycemic events in two studies [7, 8]. Thus, the advantage of one insulin over the other is unclear, except perhaps for detemir in reduction of hypoglycemic events. Further, none of these studies were conducted in a patient population of newly diagnosed children with type 1 diabetes, when the choice of basal insulin is initially made. Our study is alone in examining this particular patient population, and our results indicate that the choice of basal insulin does not influence achievement of HbA1c goal or change in HbA1c over the course of the first year.

Strengths of this study include the setting and the collection of many factors that might influence achievement of goal. The setting of the outpatient diabetes clinic is likely similar to many other clinics around the country and increases the external validity of our findings. Because the study was a retrospective chart review, it cannot be said with complete certainty that the choice of basal insulin does or does not influence glycemic outcomes. However, by including other potential confounders, the likelihood of unknown confounders was reduced, as demonstrated by our strong *R* ^2^ value. Despite this, there were still likely unknown, or known but uncontrollable, confounders that influenced the results. The number of patients in the glargine and detemir groups was not equal, which may have influenced the results and made statistical differences more difficult to detect. Other limitations include the retrospective nature of the study. As stated previously, no causality can be determined due to the design of the study. Patients were not randomized, increasing the likelihood of confounders. Chart reviews are inherently limited by the quality of documentation in the chart, and one of the variables measured was not documented thoroughly enough to perform statistical tests. Basal insulin as a percentage of total daily insulin dose was not calculated. Also, a large portion of patients were excluded from the study, including 49 for pump starts and 33 who did not have regular followup. These groups may represent potential confounders and are a large population for which this study is not applicable.

In order to obtain a decisive answer to the question of superiority of one basal insulin over another, a randomized controlled trial could be performed in this patient population. Studies similar to ours could also be performed in other clinics in order to verify the results in populations with differing baseline characteristics. Ultimately, the choice of basal insulin for a patient should be an individual one, and this study suggests that the choice does not influence glycemic outcomes at one year in a general clinic sample.

## 5. Conclusion

The only factor associated with achievement of goal HbA1c was HbA1c at diagnosis. Choice of basal insulin was not found to be a predictor of achieving HbA1c goal or of change in HbA1c over the course of the first year of diagnosis with type 1 diabetes.

## Figures and Tables

**Table 1:** Baseline characteristics.

	Glargine	Detemir	Significance
Average age (years)	10.9 ± 4.4	10.1 ± 3.6	*P* = 0.308
Male gender	58%	59%	*P* = 0.920
Caucasian ethnicity	76%	77%	*P* = 0.719
Hypothyroidism	5%	7%	*P* = 0.714
Private insurance	50%	70%	*P* = 0.055
DKA at diagnosis	24%	16%	*P* = 0.357
Flexible dosing	87%	91%	*P* = 0.514
HbA1c at diagnosis	11.8 ± 1.9	11.5 ± 2.4	*P* = 0.588
BMI percentile	45.2% ± 33.0%	43.3% ± 34.7%	*P* = 0.790
Mean daily insulin dose by unit/kg	0.70 ± 0.14	0.66 ± 0.16	*P* = 0.180
Aspart as rapid insulin	68%	100%	*P* < 0.001
Completion of education visits	87%	91%	*P* = 0.534

**Table 2:** HbA1c, change in HbA1c, achievement of goal, and total mean daily insulin dose at each time point.

	0 months	3 months	6 months	12 months
Glargine				
HbA1c	11.8 ± 1.9	6.78 ± 0.93	6.96 ± 1.66	7.98 ± 2.00
Change in HbA1c	N/A	4.97 ± 1.97	4.86 ± 2.59	3.81 ± 2.74
Achievement of goal HbA1c	0%	76%	76%	42%
Mean daily insulin dose (units/kg)	0.70 ± 0.14	0.59 ± 0.15	0.60 ± 0.17	0.69 ± 0.19
Detemir				
HbA1c	11.5 ± 2.4	7.03 ± 1.08	6.27 ± 0.93	7.30 ± 1.34
Change in HbA1c	N/A	4.51 ± 2.02	5.27 ± 2.46	4.24 ± 2.48
Achievement of goal HbA1c	7%	68%	93%	55%
Mean daily insulin dose (units/kg)	0.66 ± 0.16	0.61 ± 0.16	0.60 ± 0.18	0.66 ± 0.24
